# Molecular Characterizations and Functional Analyses of LmR2D2 in the *Locusta migratoria* siRNA Pathway

**DOI:** 10.3390/insects12090812

**Published:** 2021-09-10

**Authors:** Lu Gao, Yanli Wang, Mureed Abbas, Tingting Zhang, Enbo Ma, Shuping Xing, Kun Yan Zhu, Jianzhen Zhang

**Affiliations:** 1 Institute of Applied Biology, Shanxi University, Taiyuan 030006, China; 201713102001@email.sxu.edu.cn (L.G.); wangyanli627631821@sxu.edu.cn (Y.W.); malikmureed05@email.sxu.edu.cn (M.A.); zhangyanqiu3520@sxu.edu.cn (T.Z.); maenbo2003@sxu.edu.cn (E.M.); 2 College of Life Science, Shanxi University, Taiyuan 030006, China; xing2016@sxu.edu.cn; 3 Department of Entomology, 123 Waters Hall, Kansas State University, Manhattan, KS 66506, USA

**Keywords:** *Locusta migratoria*, dsRNA-binding protein, R2D2, RNA interference, siRNA pathway

## Abstract

**Simple Summary:**

*Locusta migratoria* is a serious agricultural pest all over the world, which devastates agriculture and challenges food security and livelihoods. Recently, RNA interference (RNAi) technology has emerged as a novel strategy for managing insect pests. However, different insect species show a significant variation in RNAi efficiency. Therefore, it is necessary to better understand the RNAi mechanism. In this study, we identified an *LmR2D2* gene in the *L. migratoria* transcriptome. After the expression of *LmR2D2* was suppressed by RNAi, we found a significantly diminished RNAi efficiency against a marker gene in *L. migratoria*. Our binding experiments further demonstrated that the LmR2D2 protein can bind double-stranded RNA (dsRNA) in vitro. On the basis of these results, we conclude that LmR2D2 is involved in the *L. migratoria* siRNA pathway.

**Abstract:**

Small interfering RNAs (siRNAs) are non-coding RNAs with a length of 21~23 nucleotides (nt) and present in almost all eukaryotes. The formation of siRNA is a highly conserved post-transcriptional gene-silencing mechanism mediated by key proteins, including Dicer2, Argonaute2 (Ago2) and R2D2. R2D2 has been identified as a double-stranded RNA (dsRNA)-binding protein and reported as an integral component of the siRNA pathway in *Drosophila*. However, the involvement of R2D2 in the siRNA pathway of *Locusta migratoria* is still unknown. In the present study, we identified an *LmR2D2* gene from the transcriptome of *L. migratoria*. It consists of a 954-bp open reading frame that encodes a protein of 318 amino acid residues. Further sequence analysis revealed that LmR2D2 possesses two tandem dsRNA-binding domains (dsRBD) at the N-terminus. Analysis of the developmental expression profile of *LmR2D2* indicated that its transcript level was stable in third-instar nymphs of *L. migratoria*, whereas the tissue-dependent expression profile exhibited high levels of expression of *LmR2D2* in the testis and ovary. When *LmR2D2* was silenced by RNAi, the RNAi efficiency against *Lmβ-tubulin* as a marker gene was significantly diminished, as indicated by the 37.7% increased *Lmβ-tubulin* transcript level. Additionally, the prokaryotic expression system was used to obtain the LmR2D2 supernatant protein. By incubating the LmR2D2 protein with biotin-dsRNA, we found that LmR2D2 can bind to dsRNA in vitro, which supports our conclusion that LmR2D2 plays an essential role in the siRNA pathway of *L. migratoria*.

## 1. Introduction

RNA interference (RNAi) is an important gene-silencing mechanism triggered by double-stranded RNA (dsRNA). This mechanism was first discovered in *Caenorhabditis elegans* by two Nobel laureates, Andrew Z. Fire and Craig C. Mello [1]. Their discovery not only provides novel approaches for analyzing gene functions but also opens new arenas for managing insect pests. Presently, scientists are considering this modern tool as an alternative to synthetic pesticides [2,3,4]. However, it is not always guaranteed that experiments involving RNAi will be successful, as a wealth of studies have demonstrated an immense variability in RNAi efficiency among different insect species, tissues, genes and the methods for dsRNA delivery [5,6,7]. Thus, to ensure an efficient insect pest management, it is necessary to have a thorough understanding about the RNAi mechanism. According to previous studies, three different RNAi pathways have been discovered in insects, including siRNA (small interfering RNA), miRNA (micro-RNA) and piRNA (piwi-interacting RNA) [8,9,10,11]. 

In the past, studies have confirmed that the siRNA pathway is mainly mediated by the ribonuclease-III-type enzymes “Dicer2” and dsRNA-binding proteins “R2D2” (the Dicer2/R2D2 complex). With the help of R2D2, Dicer2 cleaves dsRNA into 21~23-nucleotide (nt) siRNAs duplexes having a 5′ phosphate and 3′ 2-nt overhang [12,13,14]. After cleavage of the dsRNA, the siRNA is delivered to the siRNA-induced silencing complexes (siRISC) with the help of the Dicer2/R2D2 complex [15,16]. Later, with the help of helicase enzymes, the siRNA duplex is unwound into two single strands, known as sense and antisense strands. Eventually, the sense strand of the siRNA is degraded to activate RISC, while the antisense guide strand locates the complementary target mRNA. Moreover, Ago2 cleaves the target mRNA, which results in rapid silencing of the target gene [17,18,19].

In the course of purifying the enzyme that can produce siRNA in addition to Dicer2 in *Drosophila* S2 cells, a new protein containing two dsRNA-binding domains (dsRBD, R2) and interacting with Dicer2 (D2) was discovered. Delving into the functions, this protein was named R2D2 [20]. Previously, a study has demonstrated that DmR2D2 acts as a bridge between the initiation step (siRNA production) and the effector step (siRNA loading onto siRISC) of the siRNA pathway, in which R2D2 not only assists Dicer2 to stabilize dsRNA but also binds to Dicer2 to form (Dicer2/R2D2) a complex to deliver the siRNA duplex to Ago2, which is a key protein in the RISC [20]. Similarly, a study conducted by Liu et al. [15] demonstrated that the first dsRBD can bind to dsRNA by using the two dsRBD mutant proteins of DmR2D2 to perform a pull-down experiment in *Drosophila*. Furthermore, Kandasamy et al. [21] reported that the N-terminal helicase domain of DmDicer2 is more crucial in the interaction with DmR2D2 rather than the C-terminal dsRBD domain in *Drosophila*. 

Presently, our knowledge on whether the R2D2 protein affects the efficiency of RNAi in the siRNA pathway of insects is limited. To our knowledge, RNAi efficiency is quite variable among different insect species. In the Lepd-SL1 cell line of *Leptinotarsa decemlineata*, the RNAi of RNAi experiments, which are often designed to examine the potential effects of an RNAi assay against one gene on the efficiency of a subsequent RNAi assay against a different gene, have demonstrated that silencing *LdR2D2* can significantly reduce the RNAi efficiency against the *IAP* (IAP: inhibitor of apoptosis) gene, as indicated by the increased survival rate of the Lepd-SL1 cells [22]. In contrast, the expression of *BmR2D2* was found to be very low in silkworm tissues. After overexpression of *Tribolium castaneum* R2D2 (TcR2D2) in Bm5 cells, luciferase experiments revealed that the RNAi efficiency still could not be improved [23]. Using the RNAi of RNAi technique also proved that BmR2D2 does not affect the efficiency of RNAi in Bm5 cells of silkworm [24]. Thus, thorough studies are needed to determine whether R2D2 can participate in the siRNA pathway of other insect species.

The migratory locust (*Locusta migratoria*) is one of the most destructive agricultural insect pests all over the world. Over the years, this pest has been mainly managed by applications of a variety of insecticides [25,26]. The excessive use of insecticides has not only induced the development of resistance in *L. migratoria* populations but also caused environmental pollution [27], which has raised grave public concerns and compelled the experts to find safer alternatives for synthetic insecticides. Recently, RNAi has emerged as a novel tool for managing insect pests because of its high specificity, efficiency, and systemic characteristics [28]. In the present study, we, for the first time, identified the *LmR2D2* gene from the *L. migratoria* transcriptome database. By using the RNAi of RNAi approach in vivo and dsRNA binding analysis in vitro, we show that the LmR2D2 protein actively participated in the siRNA pathway of *L. migratoria*.

## 2. Materials and Methods

### 2.1. Insect Rearing

The eggs of *L. migratoria* were purchased from a locust breeding facility in Cangzhou City, Hebei Province, China, and maintained at an ambient temperature of 28 ± 2 °C, relative humidity of 40 ± 5%, and photoperiod of 14:10 h (light/dark) at the Institute of Plant Protection, Shanxi Agricultural University, Taiyuan, Shanxi, P.R. China. Locust nymphs were reared in cages (25 cm × 25 cm × 25 cm) using fresh wheat seedlings and wheat bran. Nymphs were developmentally synchronized by transferring them directly to a new cage soon after each molt.

### 2.2. Isolation and Sequencing of cDNAs Encoding LmR2D2

The complementary DNA (cDNA) of *DmR2D2* (NM_135308.2) in *D. melanogaster* was used as the query sequence to search for *LmR2D2* in the locust transcriptome database. The obtained cDNAs were translated into protein sequences using the ExPASy-translational tool (https://web.expasy.org/translate/) (accessed on 8 September 2021). Functional domains of the LmR2D2 proteins were identified using SMART (http://smart.embl.de/) (accessed on 8 September 2021). The consensus dsRBD motifs of the LmR2D2 protein were aligned with the homologous amino acid sequences of other insect species, such as *Leptinotarsa decemlineata*, *Drosophila melanogaster*, and *Bombyx mori*, by GENEDOC software. The conserved amino acid residues were identified following Tabara et al. [29]. To investigate the phylogenetic relationship among the R2D2 proteins from *L. migratoria* and other insect species, a phylogenetic tree was constructed using full-length amino acid sequences of R2D2 and the Neighbor-Joining method with 1000 bootstrap replicates in MEGA5.0 software.

### 2.3. Expression Patterns of the LmR2D2 Gene

To examine the developmental expression patterns of *LmR2D2*, one-day-old third-instar (N3D1) to five-day-old third-instar (N3D5) nymphs of *L. migratoria* were collected. To determine the tissue-specific expression patterns of *LmR2D2*, nine different tissues, including the integument, gastric caeca, foregut, midgut, hindgut, fat bodies, Malpighian tubules, testis, and ovary, were dissected from 2-day-old third-instar nymphs (N3D2) of *L. migratoria*. These experiments were repeated with four independent biological replicates. Total RNA was extracted using TRIzol reagent according to the manufacturer’s instructions (TakaRa, Tokyo, Japan) and concentrations were determined using a Nanodrop 2000 spectrophotometer (Thermo Fisher Scientific, Waltham, MA, USA). First-strand cDNA was synthesized from 1 µg of total RNA using M-MLV reverse transcriptase (TakaRa, Japan). The synthesized cDNA samples were diluted 20-fold for use as templates for reverse transcription quantitative PCR (RT-qPCR) analysis. RT-qPCR was performed using SYBR Green reagents according to the manufacturer’s instructions (Promega, Beijing, China) in a Light Cycler^®^ 480II (Roche, Basel, Switzerland). The gene-specific primers are listed in the supporting information (Table 1). The total volume of each RT-qPCR reaction was 20 µL containing 10 µL SYBR™ Green Real-time PCR Master Mix, 0.8 µL (10 µM) forward and reverse primers, a 4 µL cDNA template (20-fold diluted), and 4.4 µL nuclease-free water. The cycling conditions were 94 °C for 60 s, followed by 40 cycles of 94 °C for 5 s and 60 °C for 31 s. Melting curves were used to assess the amplification specificity in each reaction. Two technical replicates of each reaction were performed, and each experiment was replicated four times. The relative gene expression level was calculated using the 2^−Δct^ method. The elongation factors 1 alpha (*EF1α*) was used as an internal reference gene due to its stable expression both at different developmental stages and in different tissues of *L. migratoria* [30].

### 2.4. RNAi of RNAi Experiment

The primers for *LmR2D2*, *Lmβ-Tubulin*, and enhanced green fluorescent protein (*EGFP*) dsRNA syntheses were designed using the online E-RNAi web service (http://www.dkfz.de/signaling/e-rnai3/) (accessed on 8 September 2021). And synthesized by Sangon Biotech Company (Shanghai, China). The sequences of these primers are shown in Table 1. The dsRNAs were synthesized using the HiScribe^TM^ T7 High Yield RNA Synthesis Kit (NEB, Ipswich, MA, USA), confirmed by 1% agarose gel electrophoresis, quantified with a Nanodrop 2000 spectrophotometer (Thermo Fisher Scientific, USA), and were diluted to 160 ng/µL for further use. 

To determine if *LmR2D2* was involved in the *L. migratoria* siRNA pathway, we used an RNAi of RNAi strategy by examining the possible effect of RNAi against *LmR2D2* in the siRNA pathway on the efficiency of a subsequent RNAi assay against *Lmβ-Tubulin* as a marker gene. Briefly, one-day-old third-instar nymphs (N3D1) of *L. migratoria* were selected and a dose of 400 ng of dsRNA specific to *LmR2D2* or *EGFP* (control) was injected into the hemocoel through the second abdominal segment of each nymph using a microinjector. After 48 h, the same dose of ds*Lmβ-Tubulin* was re-injected into both the ds*LmR2D2-* and ds*EGFP*-injected groups. At the same time, the same dose of ds*EGFP* was re-injected into the ds*EGFP*-injected control group. Twenty-four hours after the second injection, each nymph was collected and homogenized in TRIzol for the extraction of total RNA. First-strand cDNA was synthesized from 1 μg total RNA using M-MLV reverse transcriptase (TakaRa, Japan). RT-qPCR was then performed to assess the relative expression of *LmR2D2* and *Lmβ-Tubulin*.

### 2.5. Prokaryotic Expression and Purification of LmR2D2 Protein

A pair of primers, each containing a *Bam*HI or *Not*I restriction site, was designed to facilitate a direct sub-cloning of the open reading frame (ORF) of *LmR2D2* into the pET32a vector. In brief, after cDNA was synthesized from the total RNA extracted from the ovaries of third-instar nymphs of *L. migratoria*, full-length *LmR2D2* cDNA was amplified by PCR. The pET32a vector and *LmR2D2* cDNA were simultaneously digested with *Bam*HI/*Not*I restriction enzymes and ligated with T4 DNA ligase (TaKaRa, Japan) to obtain a pET32a-LmR2D2 recombinant plasmid. After *E. coli* BL21 (DE3) competent cells were transformed by the recombinant plasmid, they were cultured overnight at 37 °C in solid Luria-Bertani (LB) medium containing ampicillin. The pET32a-LmR2D2 transformed *E. coli* colonies were picked and cultured in tubes containing liquid LB media at 37 °C. For the expression of LmR2D2 precipitated protein, the optical density reached was in the range of 0.4~0.6, the experimental group was induced with 0.5 mM isopropyl-β-D-thiogalactopyranoside (IPTG) at 37 °C for 4 h, while the control group was cultured without IPTG induction. For the expression of LmR2D2 soluble protein, the experimental group was induced with 0.2 mM IPTG at 16 °C for 20 h, while the control group was cultured without IPTG induction. Later on, samples were centrifuged at 12,000 rpm for 15 min to collect the bacterial pellet, the supernatant was discarded, and 500 μL of phosphate-buffered saline (PBS) and 5 μL (1 mM) phenylmethanesulfonyl fluoride (PMSF) were added. Sonication was performed to lyse the cells. Centrifugation was performed at 12,000 rpm for 15 min to separate the supernatant, and the pellet was resuspended in 500 μL PBS. Then, 50 μL of the supernatant or the pellet resuspension were mixed with 10 μL of 6X loading buffer and incubated at 100 °C for 10 min. The heat-treated proteins were then resolved on a 12% sodium dodecyl sulfate polyacrylamide gel electrophoresis (SDS-PAGE) gel followed by Coomassie blue staining. 

In order to obtain a sufficient amount of LmR2D2 precipitated protein, the pET32a-LmR2D2 transformed *E. coli* was inoculated into a 1 L LB medium to induce expression. After the bacterial solution was centrifuged at 8000 rpm for 15 min, the bacterial pellet was sonicated for 1 h to lyse the bacterial cells. The precipitated proteins were collected by centrifugation and dissolved with 50 mmol·L^−1^ Tris-HCl, 200 mmol·L^−1^ NaCl, and 8 mol·L^−1^ urea solution, and purified using Ni-NTA resin. Fractions were collected after gradient elution with different concentrations of imidazole (10 mM, 20 mM, 50 mM, 100 mM, 200 mM, 500 mM, and 1 M). All the fractions of protein were verified using 12% SDS-PAGE gel and the protein concentration with a single band was determined following the bicinchoninic acid (BCA) method [31]. When the concentration of the protein reached 0.5 mg/mL, the fraction was sent to the BGI Group (China) for polyclonal antibody preparation.

Meanwhile, the increased pET32a-LmR2D2 transformed *E. coli* culture was used to produce a sufficient amount of supernatant protein. After the bacterial pellet was collected by centrifugation at 8000 rpm for 15 min, 20 mL of the supernatant extraction reagent (100 mM NaCl, 20 mM Tris-HCl, 5% glycerol, pH 8.0) was added. Repeated freezing and thawing were performed, thrice in total. After the samples were sonicated for 60 min, they were centrifuged at 12,000 rpm for 30 min to collect the supernatant protein. Ni-NTA resin was used to purify the LmR2D2 supernatant protein. The concentration of the purified protein was determined by the BCA method and stored at –80 °C until further use.

### 2.6. Western Blot Analysis of LmR2D2

One-day-old third-instar nymphs (N3D1) of *L. migratoria* were injected with a dose of 400 ng of ds*LmR2D2* while the control was injected with the same dose of ds*EGFP*. Seventy-two hours after the injection, the nymphs were collected and homogenized in TRIzol reagent (TaKaRa, Japan). Relative expression of *LmR2D2* was assessed by RT-qPCR using a Light Cycler 480 instrument (Roche, Switzerland). Three biological replicates were made, and each replicate contained at least 4 nymphs. The LmR2D2 polyclonal antibody produced in mice by the BGI Group was used as the verification antibody, whereas the anti-mouse β-Tubulin monoclonal antibody (Bioworld, Visalia, CA, USA) was used as an endogenous control. For the Western blotting analysis, the total proteins were extracted by TRIzol reagent. The proteins were resolved on 12% SDS-PAGE gel and transferred onto polyvinylidene fluoride membranes (PVDF) (Millipore, Burlington, MA, USA) at 10 V for 25 min. The membrane was incubated with primary antibodies (anti-LmR2D2, 1:500; anti-β-Tubulin, 1:5000) in 5% (*w*/*v*) skimmed milk at 4 °C overnight after being pre-incubated with 5% skimmed milk at room temperature for 1 h. Goat anti-mouse (LI-COR, Lincoln, NE, USA) (1:5000) was used as the secondary antibody and incubated for 1 h at room temperature under dark conditions. LmR2D2 was detected by an odyssey infrared imaging system (LI-COR, USA).

### 2.7. Binding Assay of LmR2D2 Protein and dsRNA

Biotinylated dsRNA of *Lmβ-tubulin* (bio-ds*Lmβ-tubulin*) was synthesized using a HiScribe^TM^ T7 RNA High Yield RNA Synthesis Kit (NEB, USA) in the presence of biotin-11-uridine-5’ triphosphate (Bio-11-UTP) (Ambion, Austin, TX, USA). The synthesized bio-ds*Lmβ-tubulin* was then quantified by a Nanodrop 2000 spectrophotometer and diluted with RNase-free water to 1 μg/μL for subsequent experiments. Two micrograms of the bio-ds*Lmβ-Tubulin* was incubated with 50 μL of streptavidin beads (Thermo Fisher Scientific, USA) for 90 min at 4 °C with gentle agitation. The beads were then thrice washed with 1× binding and washing buffer (1 × BW buffer) (5 mM Tris-HCl, pH 7.5, containing 0.5 mM ethylene diamine tetra-acetic acid (EDTA), and 1 M NaCl), to remove the free dsRNA. For the treatment group, 198 μg LmR2D2 supernatant protein was incubated overnight at 4 °C with a streptavidin-bio-ds*Lmβ-Tubulin* complex, while two negative controls were set as follows: the same amount of pET32a protein incubated with streptavidin-bio-ds*Lmβ-Tubulin* complex, and the LmR2D2 supernatant protein incubated with streptavidin beads. To inhibit the degradation of protein or dsRNA, an appropriate amount of protease inhibitor cocktail (MedChemExpress, Monmouth Junction, NJ, USA) and RNase inhibitor (TakaRa, Japan) were added to the buffer. The affinity matrix was then washed thrice by a magnet with a 1X BW buffer to purify the protein that can stably bind to dsRNA. The Western blotting experiment was performed with an anti-R2D2 polyclonal antibody. 

### 2.8. Statistical Analysis

The data for *LmR2D2* expression in third-instar nymphs on different days and in different tissues were statistically analyzed by one-way ANOVA followed by Tukey’s honest significant difference (HSD) test (*p* < 0.05, SPSS software, SPSS Inc., Chicago, IL, USA). As for the detection of *LmR2D2* silencing efficiency, data were analyzed with a Student’s *t*-test. A *p* < 0.05 was considered statistically significant. Regarding the RNAi of RNAi experiment, the percentage data of the relative gene expression were transformed using arcsine square root transformation, and then the transformed data were subjected to ANOVA followed by Tukey’s HSD test.

## 3. Results

### 3.1. Domain Structure and Phylogenetic Analysis of Deduced LmR2D2 Protein

**Figure 1:**
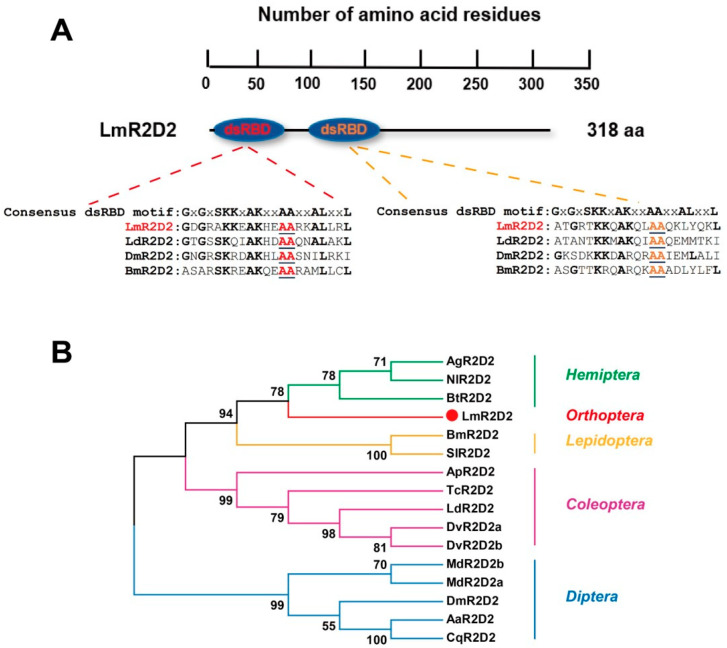
Domain structure and phylogenetic analysis of LmR2D2. (**A**) Schematic diagram of deduced domains of LmR2D2. The blue oval represents the tandem dsRNA binding domain (dsRBD). Consensus dsRBD motifs of the LmR2D2 protein were aligned with the homologous amino acid sequences of other insect species, including *L. decemlineata*, *D. melanogaster*, and *B. mori*, by GENEDOC software. The alignments show two conserved consecutive alanine (AA) residues among the species, as shown by the underlined red and orange letters. (**B**) A phylogenetic tree constructed with the neighbor-joining method of MEGA 5.0 software. Bootstrap support was based on 1000 reassembled data sets. Species names and their abbreviations, and the GenBank accession numbers for their respective R2D2 proteins, are listed in Table 2.

*LmR2D2* was identified in the transcriptome database of *L. migratoria* through the local BLAST program using the cDNA sequence *DmR2D2* (NM_135308.2) as the query sequence. Sequence analysis showed that the ORF of *LmR2D2* was 954-bp long, encoding 318 amino acid residues, which contains two dsRNA binding domains (dsRBD) at the N-terminus. The consensus dsRBD motifs of the deduced R2D2 proteins from *L. migratoria*, *L. decemlineata*, *D. melanogaster*, and *B. mori* were compared through multiple sequence alignments and revealed the conservation of two key alanine residues among the different species (Figure 1A). Phylogenetic analysis of the LmR2D2 amino acid sequences exhibited that LmR2D2 clustered with those from the insect species of Hemiptera, suggesting that LmR2D2 had a close relationship to those of the hemipteran species (Figure 1B).

### 3.2. Developmental and Tissue-Specific Expression Profiles of LmR2D2

**Figure 2:**
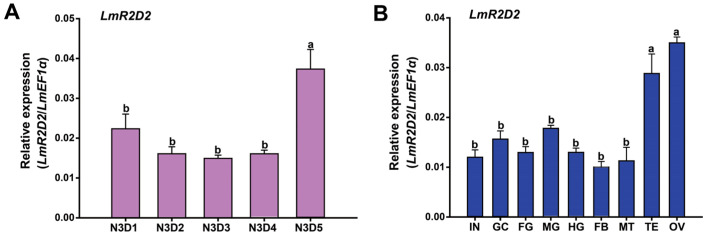
Expression of the *LmR2D2* transcript in the different stages and tissues of third-instar nymphs. (**A**) Relative transcript levels of *LmR2D2* in the whole body of N3D1-N3D5 nymphs were detected by RT-qPCR. N3D1-N3D5: the one-day-old third-instar nymphs to the five-day-old third-instar nymphs. (**B**) Relative transcript levels of *LmR2D2* in different tissues of N3D2 nymphs were determined by RT-qPCR, which include the integument (IN), gastric caeca (GC), foregut (FG), midgut (MG), hindgut (HG), fat body (FB), Malpighian tubules (MT), testis (TE), and ovary (OV). All data are reported as the mean ± standard error of four independent biological replicates. Different letters above the bars represent significant differences (*p* < 0.05, Tukey’s HSD test; *n* = 3).

The relative expression level of *LmR2D2* was examined in *L. migratoria* nymphs from N3D1 to N3D5 by RT-qPCR. Our results demonstrated stable expression of *LmR2D2* in the one-day to four-day-old nymphs but significantly increased expression in the five-day-old nymphs of the third stadium (Figure 2A). On the other hand, the relative expression levels of *LmR2D2* were determined in nine different tissues (integument, gastric caeca, foregut, midgut, hindgut, fat bodies, Malpighian tubules, testis, and ovary) from *L. migratoria* N3D2 nymphs. Our results showed the highest expression of *LmR2D2* in the testis and ovary among all the tissues investigated (Figure 2B).

### 3.3. Effect of RNAi against LmR2D2 on RNAi Efficiency

**Figure 3:**
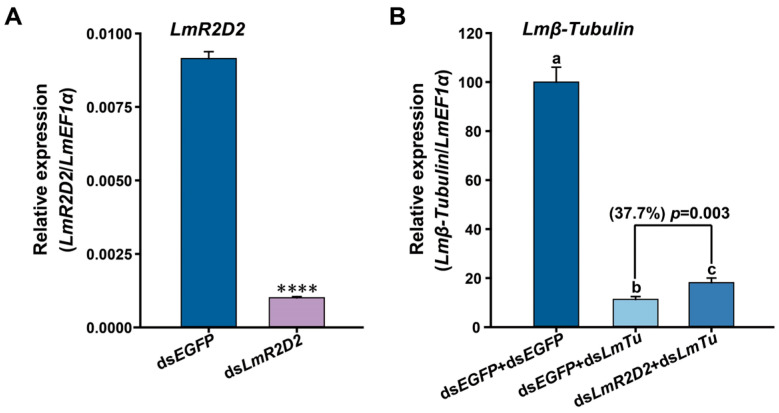
Effects of *LmR2D2* expression suppression on RNAi efficiency in third-instar nymphs. (**A**) Relative transcript levels of *LmR2D2* following injection of gene-specific dsRNA (mean ± SE, n ≥ 20; **** denotes *p* < 0.0001). (**B**) Relative transcript levels of *Lmβ-Tubulin*. The third-instar *L. migratoria* nymphs were injected with ds*LmR2D2* or ds*EGFP*, a second injection of ds*Lmβ-Tubulin*(ds*LmTu*), or ds*EGFP* was performed 48 h later. The gene-silencing efficiency of the *Lmβ-Tubulin* reporter gene was detected by RT-qPCR. Different letters on the bars indicate a statistically significant difference based on an ANOVA followed by Tukey’s HSD multiple comparison test (*p* < 0.05).

To determine the possible contribution of *LmR2D2* to RNAi efficiency in *L. migratoria*, the RNAi of RNAi strategy was applied. Our results showed that *LmR2D2* transcript level was significantly reduced after the injection of ds*LmR2D2* (Figure 3A), and the suppression of *LmR2D2* prior to the injection of ds*Lmβ-Tubulin* diminished the silencing efficiency of *Lmβ-Tubulin* by 37.7% (Figure 3B).

### 3.4. Expression and Purification of Recombinant LmR2D2 Protein

**Figure 4:**
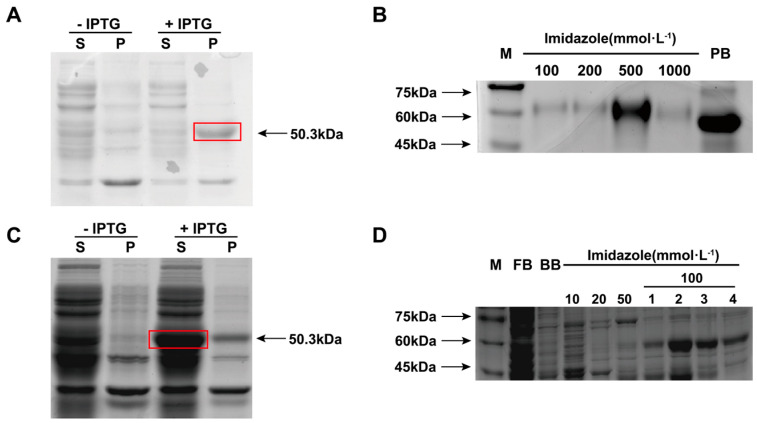
Prokaryotic expression (**A**,**C**) and analyses of recombinant LmR2D2 protein (**B**,**D**). LmR2D2 was expressed in the *E. coli* BL21 (DE3) bacterial strain transformed with pET32a-LmR2D2 plasmid and analyzed with 12% SDS-PAGE. Two different expression conditions were tested, as shown in Panel A: absence or presence of 0.5 mM IPTG at 37 °C for 4 h, and in Panel C: absence or presence of 0.2 mM IPTG at 16 °C for 20 h. In both panels, S and P represent the soluble (supernatant) and precipitated fractions of the LmR2D2 samples, respectively. The red boxes on the gel images indicate a 50.3-kDa LmR2D2 protein predominately expressed in the precipitated (**A**) and soluble (**C**) fractions. After the expression, the protein was purified from the precipitated (**B**) and soluble (**D**) fractions using a Ni-NTA purification system and analyzed with 12% SDS-PAGE. In Panel D, the numbers 1–4 under 100 represent four fractions of the protein eluted by 100 mmol·L^−1^ imidazole. M: molecular size markers; PB: protein before purification; FB: flow-through buffer; BB: binding buffer.

Using a prokaryotic expression system, the LmR2D2 recombinant protein was successfully expressed in the precipitate. The size of the protein was measured to be about 50.3 kDa, which was consistent with the predicted molecular mass based on the amino acid compositions of the deduced LmR2D2 protein and the His-tag (Figure 4A). Meanwhile, the LmR2D2 recombinant protein was also expressed to the supernatant, with the same molecular mass (Figure 4C). To prepare the LmR2D2 mouse polyclonal antibody, a large amount of LmR2D2 precipitated protein was expressed. Our results showed that LmR2D2 precipitated protein had the highest concentration and purity form the 500-mmol·L^−1^ imidazole eluent. The LmR2D2 concentration was 0.5 mg/mL, as measured by the BCA method (Figure 4B). Further, to demonstrate whether the LmR2D2 protein can bind dsRNA in vitro, a large amount of LmR2D2 protein was expressed in the supernatant. Our results showed that the LmR2D2 supernatant protein had a high concentration, with a major band in the 100-mmol·L^−1^ imidazole eluent. The concentration in the third fraction eluted by 100 mmol·L^−1^ imidazole was 1.65 mg/mL (Figure 4D).

### 3.5. Effect of RNAi Targeting LmR2D2 on Its mRNA and Protein Levels

**Figure 5:**
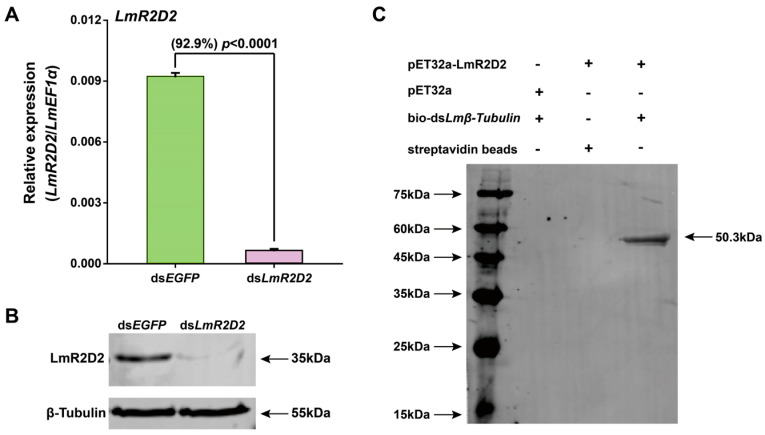
Binding analysis of the LmR2D2 protein with dsRNA in vitro. (**A**,**B**) LmR2D2 polyclonal antibody verification. One-day-old third-instar nymphs (N3D1) were injected with ds*LmR2D2* and homogenized at 72 h after injection. The total RNA and total protein were extracted using the TRIzol method. Panel A shows the silencing efficiency of *LmR2D2*, as determined by RT-qPCR. The data are reported as the mean ± standard error of three independent biological replicates. Data were statistically analyzed using Student’s *t*-test. Panel B represents the results from the Western blotting analysis for detecting the LmR2D2 protein after the *LmR2D2* gene was silenced by RNAi. The LmR2D2 polyclonal antibody was used for detecting the LmR2D2 protein, and the β-Tubulin monoclonal antibody was used as the loading control. (**C**) LmR2D2 protein enriched to the streptavidin-bio-ds*Lmβ-Tubulin* complex was separated by SDS-PAGE, and detected by Western blotting using the LmR2D2 polyclonal antibody.

To examine the efficiency of RNAi targeting *LmR2D2* and validate the specificity of the LmR2D2 polyclonal antibody, we used RT-qPCR and Western blotting to examine the relative *LmR2D2* mRNA and its protein levels, respectively. Our results showed that RNAi decreased the transcript level of *LmR2D2* by 92.9% compared with the control (Figure 5A). Western blotting analysis showed a significant reduction in the LmR2D2 protein level (Figure 5B), indicating that ds*LmR2D2* can effectively suppress the expression of LmR2D2 at both the mRNA and protein levels. Further, these results also indicated that the LmR2D2 polyclonal antibody can specifically recognize the LmR2D2 protein in *L. migratoria*.

### 3.6. Binding of LmR2D2 Protein to dsRNA In Vitro

To test our hypothesis that LmR2D2 can efficiently bind to dsRNA, we performed an in vitro binding assay. Our results clearly showed that the LmR2D2 antibody can strongly interact with a 50.3 kDa protein after the LmR2D2 protein was incubated with a streptavidin-bio-ds*Lmβ-Tubulin* complex. However, the LmR2D2 antibody did not interact with the control sample, indicating that LmR2D2 can bind to dsRNA in vitro (Figure 5C).

## 4. Discussion

### 4.1. Molecular Characterization of R2D2

In the present study, a cDNA sequence of *LmR2D2* was identified from the *L. migratoria* transcriptome database. Sequence analysis of *LmR2D2* showed that it possessed a 954-bp ORF and putatively encoded a protein of 318 amino acid residues. Protein structures of LmR2D2 predicted by the SMART domain analysis tool revealed that the protein contained two dsRBD domains at its N-terminus. Consensus dsRBD motifs of the deduced R2D2 proteins from *L. migratoria*, *L. decemlineata*, *D. melanogaster*, and *B. mori* were compared using multiple sequences alignments. Our results showed that the two alanine residues were highly conserved among all these insect species, suggesting that R2D2 can bind dsRNA or siRNA. In previous reports, a pull-down experiment and gel-shift assay indeed proved that two highly conserved alanine (A) residues of dsRBD motifs in DmR2D2 were very important for dsRNA and siRNA binding in *Drosophila* [15,20]. Thus, our results are consistent with previous studies in *Drosophila.*

Phylogenetic analysis showed that LmR2D2 was closely related to the R2D2 proteins of hemipteran insects, including *Bemisia tabaci*, *Aphis glycines*, and *Nilaparvata lugens*. Tissue-specific expression analysis of the *LmR2D2* gene showed the highest expression in the ovary and testis, indicating that this gene may play a fundamental role in the reproductive processes of *L. migratoria*. Previously, research conducted by Kalidas et al. [32] showed a significant reduction in fertility of the DmR2D2 mutants in *Drosophila*, which affirms the crucial role of DmR2D2 in the ovary. 

### 4.2. Involvement of LmR2D2 in siRNA Pathway

Several past studies have reported that R2D2 is a dsRNA-binding protein that may function as a cofactor of Dicer2, implying the role of R2D2 in the siRNA pathway [15,20]. However, it is unknown whether LmR2D2 plays the same role in the *L. migratoria* siRNA pathway. By using the RNAi of RNAi strategy, our results revealed that the suppression of *LmR2D2* expression can significantly diminish the RNAi-mediated silencing efficiency against the target gene *Lmβ-Tubulin*, as compared with the control (ds*EGFP* + ds*Lmβ-Tubulin*). Our findings are in agreement with Yoon et al. [22], who reported an efficient suppression of the target gene *LdR2D2* in the siRNA pathway in the Lepd-SL1 cell line in *L. decemlineata*. When ds*IAP* (IAP: inhibitor of apoptosis) was injected, a significant increase in the survival rate of cells was observed, indicating that R2D2 can affect the RNAi efficiency. In contrast, some studies have reported that, in lepidopterans, such as *B. mori*, the expression of *BmR2D2* in different tissues was very low [23] and the RNAi of RNAi experiments also entailed that BmR2D2 had no effect on RNAi efficiency in the silkworm Bm5 cell line [24]. Such a difference could be due to the variation in insect species, in which R2D2 as a siRNA pathway core protein might have different properties in responding to exogenous dsRNA. 

**Figure 6:**
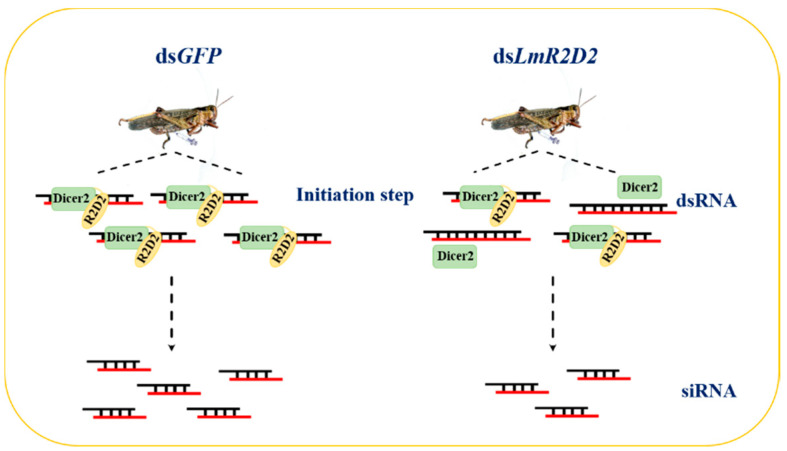
Model illustrating the involvement of LmR2D2 in the siRNA pathway. According to our results from this study, it is proposed that LmR2D2 is an essential protein in the initiation step of the siRNA pathway mediated by exogenous dsRNA in *L. migratoria*. LmR2D2 assists LmDicer2 to recruit dsRNA, which is processed to siRNA by LmDicer2. If the expression of the *LmR2D2* gene is suppressed by RNAi-mediated gene silencing, it ultimately reduces the translation of *LmR2D2* mRNA into the LmR2D2 protein. The reduced amount of LmR2D2 protein decreases the number of dsRNA being recruited to LmDicer2, which subsequently decreases the RNAi efficiency in *L. migratoria*.

To validate the involvement of LmR2D2 in the siRNA pathway, the LmR2D2 supernatant protein was obtained by using a prokaryotic protein expression system. When the LmR2D2 supernatant protein was incubated with a streptavidin-bio-ds*Lmβ-Tubulin* complex in vitro, the Western blotting analysis showed that the LmR2D2 antibody can interact with a 50.3-kDa protein, which entailed that the dsRBD domain of the LmR2D2 protein can interact with the backbone of dsRNA. However, the control pET32a protein without the dsRBD domain showed no signal, suggesting that pET32a does not interact with dsRNA. In *Drosophila*, it has also been demonstrated that DmR2D2 can bind to dsRNA through a pull-down experiment [15]. In addition, a previous study on RDE-4 in *C. elegans* (homologous to the *Drosophila* RNAi protein R2D2) also showed that RDE-4 can bind dsRNA [29]. Based on our current results, we propose that LmR2D2 plays an important role in the initiation step of the siRNA pathway mediated by exogenous dsRNA in *L. migratoria*, where LmR2D2 can assist LmDicer2 to recruit dsRNA and process it to siRNA. When the expression of the *LmR2D2* gene is suppressed by RNAi-mediated gene silencing, the production of the LmR2D2 protein is ultimately decreased. The reduced amount of LmR2D2 protein subsequently reduces the amount of dsRNA being recruited to LmDicer2, which finally affects the RNAi efficiency against an RNAi target gene (Figure 6). Our study represents the first research effort in elucidating the role of LmR2D2 in the siRNA pathway in an agriculturally important orthopteran insect pest.

## 5. Conclusions

In the current study, we identified a full-length cDNA sequence of *LmR2D2* from the *L. migratoria* transcriptome database. The LmR2D2 protein contains two dsRBD domains at its N-terminus. Multiple sequence alignments showed that the two alanine residues of the consensus dsRBD motifs in the R2D2 of *L. migratoria* were highly conserved in different insect species. Further experiments using the RNAi of RNAi strategy in vivo and the binding experiments using western blotting analysis in vitro validated that LmR2D2 played an important role in the initiation step of the siRNA pathway mediated by exogenous dsRNA in *L. migratoria*. Our study is expected to facilitate mechanistic studies on the siRNA pathway in *L. migratoria* and other insect species. 

## Figures and Tables

**Table 1:** Primers for full-length validation, PCR amplification, and dsRNA synthesis.

Application of Primers	Primer Names	Primer Sequence (5′–3′)	Product (bp)
Full-length verification	*LmR2D2*-F	ACATGAATCAGAAGACACCAGTGTCAGT	954 bp
	*LmR2D2*-R	TTCATCCTGGACGCCTTCCTGATGAGAG	
RT-qPCR Analysis	*LmR2D2* RT-F	TCCGTTGGTTTGCTGATTGA	103 bp
	*LmR2D2* RT-R	TGGTGAACTGCTTGGCGTGT	
	*EF1α* RT-F	AGCCCAGGAGATGGGTAAAG	155 bp
	*EF1α* RT-R	CTCTGTGGCCTGGAGCATC	
	*Lmβ-Tubulin* RT-F	GAAATGGAGTTCACGGAAGC	109 bp
	*Lmβ-Tubulin* RT-R	CTTGCTCCTCATCAAACTCG	
dsRNA synthesis	*EGFP* T7-F	taatacgactcactatagggGACGTAAACGGCCACAAGTT	496 bp
	*EGFP* T7-R	taatacgactcactatagggTGTTCTGCTGGTAGTGGTCG	
	*LmR2D2* T7-F	taatacgactcactatagggATCAAAAGATTGCCACAGGC	493 bp
	*LmR2D2* T7-R	taatacgactcactatagggGCATTCTGCATAGCCTCCTC	
	*Lmβ-Tubulin* T7-F	taatacgactcactatagggAGGCCACTACACAGAGGGTG	401 bp
	*Lmβ-Tubulin* T7-R	taatacgactcactatagggTGACGCCAGACATGGTAAGA	

**Table 2:** Insect species and GenBank accession numbers of the deduced proteins used for the phylogenetic tree (Figure 1B) created in this study.

Species	Gene Name	GenBank Accession No.
*Aphis glycines*	*AgR2D2*	JX870426.1
*Nilaparvatalugens*	*NlR2D2*	KC316044.1
*Bemisiatabaci*	*BtR2D2*	KF192312.1
*Locusta migratoria*	*LmR2D2*	MZ313537
*Bombyx mori*	*BmR2D2*	AB566385.1
*Spodoptera litura*	*SlR2D2*	KF717086.1
*Agrilusplanipennis*	*ApR2D2*	KP036494.1
*Triboliumcastaneum*	*TcR2D2*	NM_001134953.1
*Drosophila melanogaster*	*DmR2D2*	NM_135308.2
*Aedes aegypti*	*AaR2D2*	KJ598053.1
*Culex quinquefasciatus*	*CqR2D2*	XM_001861911.1
*Mayetiola destructor*	*R2D2a*	AFX89026.1
*Mayetiola destructor*	*R2D2b*	AFX89027.1
*Diabrotica virgifera virgifera*	*R2D2a*	XP_028140225.1
*Diabrotica virgifera virgifera*	*R2D2b*	XP_028148994.1

## Data Availability

Sequence data are available in a publicly accessible repository: GenBank accession numbers are shown in Table 2.
